# Annotations

**Published:** 1893-05-27

**Authors:** 


### ANNOTATIONS.

**"Washed Daily on the Allotment System."** WE have not, as yet, been able to make a "collection" of "nurses' curiosities," but the facts are decidedly accumulating. By way of example, the following appears to us to be unsurpassed. A series of questions were published in THE HOSPITAL, and written answers were requested on the subject of the nursing of bronchitis patients. Many answers verging on the "funny" were received, but this was the climax of them all: In bronchitis, says Nurse ——, "the patient should be washed daily on the allotment system, under a hot blanket." It is a pity to spoil so delightful a statement of nursing principles by anything so commonplace as common-sense. And yet one cannot but perceive what a vista of possible confusions and failures to grasp realities is opened out by a single sentence of this kind. What "washing on the allotment system" may mean it is impossible even to conjecture, but it is evident that the nurse who lays down this rule for us has a great sense of the importance, and still more of the mysteriousness, of her calling. But we would fain impress upon nurses, as upon doctors, that the way to the uncommon is through the common; and she who learns thoroughly the obvious and the easy is making the best possible preparation for learning the more complicated and the more difficult. We want cultivated, but not confused nurses; practical, but not priggish guardians of the sick. When shall we all learn that "well" is better than "much"? A farthing candle held close to the book is of more service in a dark night than Venus or Mercury away in the distant sky.

**Is Cancer Increasing?** WE were enabled last week, by the courtesy of the Council of the Royal Society, to publish an abstract of the paper communicated to the Society on this subject by Messrs. George King, F.I.A., and Arthur Newsholme, M.D. It is clear that the question of the increase or otherwise of cancer among the most civilised races is one of prime importance, for the fact alone of its certain increase would go far to discredit civilisation, and to establish the superior merits of "a state of nature," as it is called. It requires no very great inductive capacity to see that if cancer is increasing among us we may now, or soon, look with certainty for an increase of other diseases which depend more or less either upon impaired vitality or upon the mixed inherited diatheses of civilisation. Corresponding accurately with such a physical condition we may look for a degradation, more or less uniform, of the mental powers. In short, a decisive increase of cancerous disease and of cancer mortality may be regarded as the announcement of the beginning of a downward stage in national history, and a preliminary to permanent national degradation of physique and character. Messrs. King and Newsholme have extended their inquiries over a wide range. The Registrar-General's statistics for England, Scotland, Ireland, and Wales have furnished their principal data; but, in addition, they have made exhaustive researches, for purposes of comparison, into the cancer history, as we may call it, of one of the largest and oldest of British insurance societies, the Scottish Widows, and also into the official cancer records of Frankfort-on-the-Main. The investigators have announced with some decision, that they do not agree with the recent conclusion that there is an actual increase of cancer and cancer mortality: on the contrary, they hold, with earlier inquirers, that more competent diagnosis is sufficient to account for the apparent increase. Messrs. King and Newsholme, whether their conclusions be accurate or not, have many striking facts to urge in favour of their contention. Among these is the fact that Ireland shows a lower percentage of cancer than any other portion of the United Kingdom. The investigators do not believe that there is actually less cancer in Ireland, but that competent medical skill is less general, and that diagnosis is therefore less efficient. Similarly they find that at Frankfort, where the cancer cases are so classified as to enable the investigators to divide all the cases into the two groups of "accessible" and "inaccessible," it is found that there has been no increase at all, in proportion to the growth of the population, of cases of "accessible" cancer. These two sets of facts, and especially the latter set, are very striking, and point decidedly in the more hopeful direction of at least a stationary condition of concerous diseases and cancer mortality. The abstract which we have been enabled by the courtesy of the Royal Society to publish, is deeply interesting, and we venture to suggest to the Society that the medical profession and the public would be grateful for the publication of the orignal communication in full.

**George Smith: the Man or his Monument?** "WE think it wiser to help a man while he is living and at work than to erect a monument because of his noble deeds after he is dead." This is a sensible general proposition: let us couple it with another from the same source: George Smith "is now in his sixty-third year; he is in much need of help, and is worthy of it." Almost everybody knows who George Smith of Coalville is, what he has done, and what are his consequent claims upon the gratitude of sensible people. But for those who do not, we will give two or three sentences of biography. George Smith has been devoted entirely to philanthropic work for twenty years. He gave up his secular work twenty years ago, and with it his income of £450 per annum, not for the cloister but for the cry of the children. Among other things he has been more than a father to 70,000 British children of the Brickfield and Canal Boat class. To his determined and persevering resolution it was owing that Parliament framed the special and beneficent measures known as the "Brickfield" and the "Canal Boats" Acts. At present Mr. Smith has in hand a similar work, which will secure the education, and the moral and religious training of 50,000 gipsy and other children, who spend their lives in country roads and lanes in caravans. This proposed measure is called the "Moveable Dwellings Bill." To be the protecting genius and good angel of 120,000 of the most neglected, untaught, illused, and unhappy children in the world is no small honour. But if the honour is great the responsibility is almost greater still. To have this responsibility and lack money for urgent daily needs is a condition of things that none but the bravest and most duty-loving would so much as think of facing. It is clear that Mr. Smith must be relieved of the anxiety of providing for his daily needs if his unique work for this army of uncared for children is to be carried on till the end of his life. It is a great satisfaction to us to know that a committee has been formed which is almost as unique as Mr. Smith and his work. The Committee consists of representative religious teachers of all denominations, including Archdeacon Sinclair on the one hand, and the Rev. J. Treves, President of the Primitive Methodist Conference, on the other. It is proposed to raise three thousand pounds. Mr. F. D. Allcroft leads off with £50, and Mr. Edwin Lawrence follows with another £50. That is the way to begin. We wish we could persuade fifty readers of THE HOSPITAL to hurry to the front with £50 each, and so to raise the money out of hand. Mr. Edwin Lawrence, of 10, Kensington Palace Gardens, London, W., is the treasurer, and will receive subscriptions.

